# A prospective longitudinal study of Pasireotide in Nelson’s syndrome

**DOI:** 10.1007/s11102-017-0853-3

**Published:** 2018-01-08

**Authors:** Eleni Daniel, Miguel Debono, Sharon Caunt, Constantine Girio-Fragkoulakis, Stephen J. Walters, Scott A. Akker, Ashley B. Grossman, Peter J. Trainer, John Newell-Price

**Affiliations:** 1 Department of Oncology and Metabolism, The Medical School, University of Sheffield, Beech Hill Road, Sheffield, S10 2RX UK; 2 Sherwood Forest Hospitals, Nottinghamshire, NG17 4JL UK; 3 School of Health and Related Research and NIHR Research Design Service Yorkshire and the Humber, University of Sheffield, Regent Court, 30 Regent Street, Sheffield, S1 4DA UK; 4 St Bartholomew’s Hospital, West Smithfield, London, EC1A 7BE UK; 5 University of Oxford and Oxford Centre for Diabetes, Endocrinology and Metabolism Churchill Hospital, Oxford, OX3 7LE UK; 6 The Christie NHS Foundation Trust, Manchester Academic Health Science Centre, University of Manchester, Wilmslow Road, Manchester, M20 4BX UK

**Keywords:** Nelson’s, Pasireotide, Medical therapy, Corticotroph pituitary adenoma

## Abstract

**Purpose:**

Nelson’s syndrome is a challenging condition that can develop following bilateral adrenalectomy for Cushing’s disease, with high circulating ACTH levels, pigmentation and an invasive pituitary tumor. There is no established medical therapy. The aim of the study was to assess the effects of pasireotide on plasma ACTH and tumor volume in Nelson’s syndrome.

**Methods:**

Open labeled multicenter longitudinal trial in three steps: (1) a placebo-controlled acute response test; (2) 1 month pasireotide 300–600 μg s.c. twice-daily; (3) 6 months pasireotide long-acting-release (LAR) 40–60 mg monthly.

**Results:**

Seven patients had s.c. treatment and 5 proceeded to LAR treatment. There was a significant reduction in morning plasma ACTH during treatment (mean ± SD; 1823 ± 1286 ng/l vs. 888.0 ± 812.8 ng/l during the s.c. phase vs. 829.0 ± 1171 ng/l during the LAR phase, p < 0.0001). Analysis of ACTH levels using a random intercept linear mixed-random effects longitudinal model showed that ACTH (before the morning dose of glucocorticoids) declined significantly by 26.1 ng/l per week during the 28-week of treatment (95% CI − 45.2 to − 7.1, p < 0.01). An acute response to a test dose predicted outcome in 4/5 patients. Overall, there was no significant change in tumor volumes (1.4 ± 0.9 vs. 1.3 ± 1.0, p = 0.86). Four patients withdrew during the study. Hyperglycemia occurred in 6 patients.

**Conclusions:**

Pasireotide lowers plasma ACTH levels in patients with Nelson’s syndrome. A longer period of treatment may be needed to assess the effects of pasireotide on tumor volume.

Trial registration: Clinical Trials.gov ID, NCT01617733

**Electronic supplementary material:**

The online version of this article (10.1007/s11102-017-0853-3) contains supplementary material, which is available to authorized users.

## Introduction

Nelson’s syndrome is a very challenging condition that can develop following bilateral adrenalectomy (BLA) for Cushing’s disease (CD), and is due to the development of a progressive tumor of the corticotroph cells in the pituitary [1]. It occurs in up to 30% of patients with CD undergoing bilateral adrenalectomy [2] although progression of the size of a corticotroph tumor as assessed by MRI is more common and is detected in 50% of patients within 10 years of BLA for CD [3, 4]. The corticotroph tumor may be small in some cases but may also be extensive and locally invasive in others; patients can present with mass effects, headache, visual field defects, and external ophthalmoplegia [5, 6]. The hallmarks of the syndrome are skin hyperpigmentation and high plasma adrenocorticotropic hormone (ACTH) levels that reflect the activity of the tumor and are used for monitoring [7]. Treatment of Nelson’s is restricted to pituitary surgery and radiotherapy only when there is an amenable anatomic target and the patient’s condition allows [8–10]. In many patients with Nelson’s syndrome these conditions are not met; the levels of ACTH continue to rise, the symptoms persist and there are limited treatment options.

There is currently no medical therapy that can consistently reduce plasma ACTH levels and corticotroph tumor growth, and there is a real need for an effective medical management for Nelson’s syndrome. The anti-epileptic sodium valproate was frequently used in the past with disappointing or variable results [11–16] and dopamine agonists such as cabergoline only occasionally result in satisfactory response [17–20]. Peroxisome proliferator-activated receptor gamma (PPARγ) agonists such as rosiglitazone have also been studied: one report showed biochemical response in two out of three patients, but one of these subsequently escaped [21]. Our group previously showed that even high doses of rosiglitazone (12 mg/day) do not reduce plasma ACTH levels [5]. Temozolomide is a medical treatment for aggressive pituitary tumors, but is associated with significant toxicity limiting its use [22].

Pasireotide exerts its pharmacologic effects by binding and activating multiple somatostatin receptor subtypes (1, 2, 3, and 5). In vitro experiments have shown that pasireotide inhibits ACTH secretion in cultured corticotroph adenoma cells [23] and prospective clinical trials have proved effectiveness in lowering cortisol levels in patients with active Cushing’s disease [24–26]. More recently, pasireotide LAR was used to treat a patient with an invasive corticotroph tumor resulting in clinical improvement, and reductions in tumor size and plasma ACTH levels [27].

In light of these data we have performed a prospective multicenter clinical study aiming to investigate the effects of pasireotide on circulating plasma ACTH and tumor size in patients with Nelson’s syndrome. In particular, the study was structured to assess: (1) the acute effects of pasireotide on circulating levels of plasma ACTH after a single 600 μg s.c. injection, and whether this would allow prediction of individual longer-term response, (2) the effects of 4-week of pasireotide s.c. on circulating plasma ACTH, (3) the effects of pasireotide LAR given monthly for 6 months on circulating plasma ACTH, and (4) the effect of pasireotide s.c. and LAR on tumor volume.

## Methods

### Study design

**Fig. 1:**
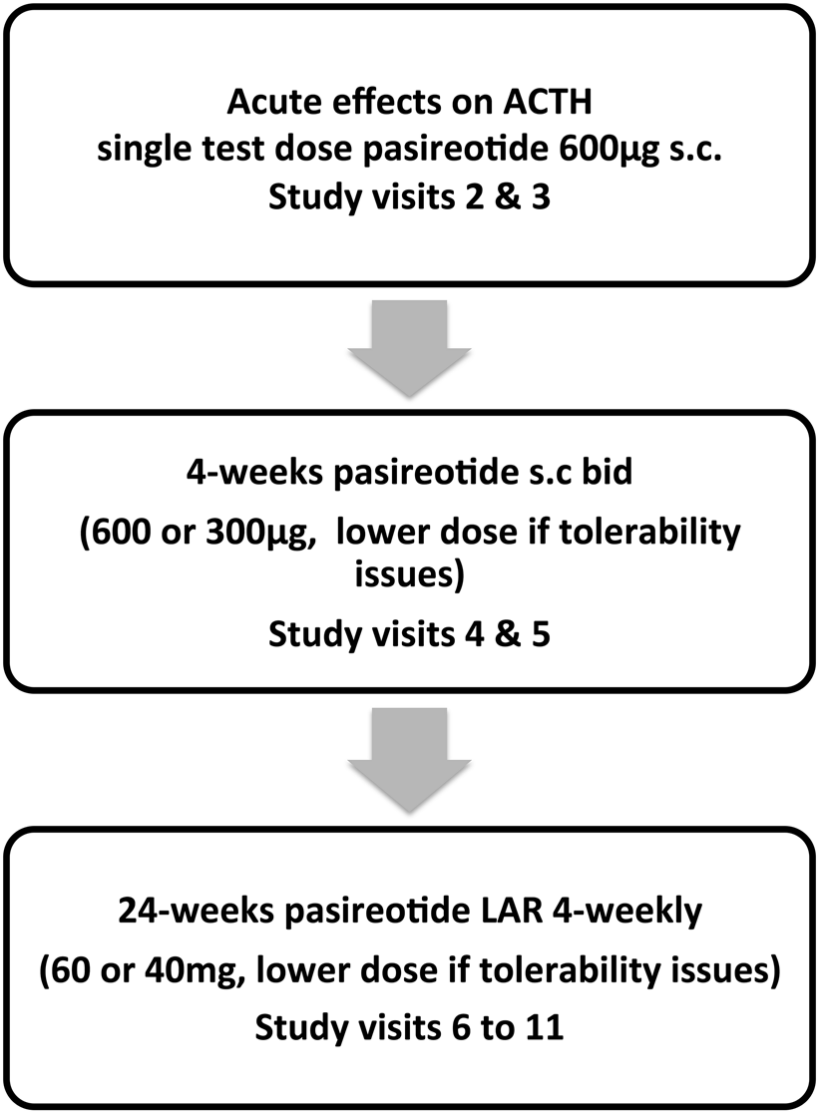
Pasireotide treatment in Nelson’s syndrome: study design

This was an open labeled longitudinal trial over a 31-week period conducted in four tertiary centers in England, UK. As there is currently no alternative treatment for Nelson’s syndrome, no control group was used. There were three parts to the study. Initially, an acute response of plasma ACTH to pasireotide was assessed in a placebo-controlled randomized single-blinded crossover intervention where patients received either a test dose of 600 μg pasireotide s.c. or an equivalent volume of saline s.c. whilst omitting their glucocorticoid treatment to establish if an acute response predicts future efficacy. In the second part of the study, patients received short-term (4-week) open label treatment with pasireotide twice-daily s.c. (600 μg b.d. or 300 μg b.d. if dose reduction due to tolerability was necessary). In the last part of the study patients had long-term open label treatment with pasireotide LAR 60 mg (or 40 mg if reduced for tolerability) every 28 days for 24 weeks (Fig. 1).

### Study endpoints

#### Primary endpoint

Early morning plasma ACTH sampled before (0 h), and 2 h after morning glucocorticoid (GC) replacement during 4 weeks of pasireotide s.c. 1200 μg/day (or 600 μg/day if reduced for tolerability issues) compared with levels at these respective time points found at baseline and after 6-months depot pasireotide LAR i.m. every 28 days. The response criteria were defined according to the fall in plasma ACTH prior to first morning dose of GC or the fall in plasma ACTH 2 h after the morning dose of GC. Complete success was defined as a fall in pre-GC plasma ACTH > 400 ng/l, or fall of > 200 ng/l 2 h after GC; partial success a fall in pre-GC plasma ACTH < 399 ng/l > 200 ng/l, or 2 h after GC < 199 ng/l > 100 ng/l; and not successful a fall in pre-GC plasma ACTH < 199 ng/l, or 2 h after GC < 99 ng/l. The baseline value of the pre-GC ACTH was the mean of 4 values (from visits 1 to 4). The baseline of the post-GC ACTH was the value from visit 1 (screening visit).

#### Secondary endpoints

(1) Plasma ACTH before and at 2, 3, 4, 5, and 6 h after an acute single dose of 600 μg pasireotide or saline. (2) Changes in tumor volume at the end of the study determined by MRI. (3) Changes in skin pigmentation at the end of the study period compared with pre-treatment. (4) Change in HbA1c, fasting insulin and glucose levels during pasireotide s.c. and LAR treatment. (5) Tolerability and safety of pasireotide.

### Patients

Patients with Nelson’s syndrome were eligible to take part in this study. All patients gave informed consent and the study was approved by the UK Health Research Authority (ref 10/H1005/53). The inclusion criteria were: male or female patients aged 18–80 years with signs, symptoms and biochemistry consistent with Nelson’s syndrome and a negative pregnancy test (where applicable). The exclusion criteria were: (1) pituitary radiotherapy within the last year prior to study entry, (2) recent significant deterioration in visual fields or other neurological signs related to tumor mass requiring surgery, (3) severe liver disease, (4) symptomatic cholelithiasis, (5) clinically significant abnormal laboratory values, (6) a QTcF interval measured on the EKG > 480 ms, (7) pregnancy or lactation, (8) recent (last 6 months) history of alcohol or drug abuse, (9) concurrent administration of investigational drug for another study, (10) history of non-compliance, or inability to complete the entire study for any reason. Skin pigmentation was assessed at screening, at the start and at the end of pasireotide LAR treatment. Patients received no previous medical treatment for NS; patient characteristics are provided in the Supplementary Table.

### Measurements

#### Imaging

Gadolinium-enhanced MRI of the pituitary was performed at the participating centers before and after treatment to assess the tumor volume. A blinded radiologist assessed the scans using standard volumetric techniques [28–30]. Abdominal USS was performed at screening and at the end of the study to assess for the presence of cholelithiasis.

#### Skin pigmentation

An assessment of the pigmentation by the attending physicians and medical photographs of participants were performed at screening, at the start, and at the end of pasireotide LAR treatment. The photographs of all participants were collected and analyzed centrally.

#### Assays

Fasting insulin and ACTH samples were collected and analyzed at the central Clinical Chemistry laboratory. Insulin was measured with the Roche electrochemiluminescence immunoassay on a cobas e602 module (reference range 17.8–173 pmol/l, CV 1.8 and 2.5% at values of 121 and 2062 pmol/l). ACTH was measured by chemiluminescent immunometric assay on the Siemens Immulite 2000 analyzer (reference range < 46 ng/l at 9 a.m. and < 15 ng/l at midnight, CV 5.56 and 6.94% at values of 26.2 and 382 ng/l).

### Statistical analysis

#### Sample size

A target of 17 patients was calculated taking into account the variability of ACTH levels and a 13% dropout rate which was recorded for a pasireotide phase 2 study [31]. Accounting for a within person variability of ACTH levels of approximately 400 ng/l for the pre-GC dose and 250 ng/l post-GC dose, 15 patients were needed to detect a clinically significant change of 200 ng/l with a power of 80% with 5% significance and a further two patients to cover possible dropout [5].

Statistical analysis was performed using GraphPad (6.0d GraphPad Software, La Jolla California USA), SPSS v22 (IBM Corp., Armonk, NY) and STATA (StataCorp., College Station, TX: StataCorp LP). The main aim of the analysis was to establish whether ACTH levels change over time after pasireotide therapy. ACTH levels at 0 h (before morning glucocorticoid dose) and 2-post GC dose, were compared before onset of pasireotide treatment (‘baseline’), during s.c. and LAR pasireotide treatment using the Kruskal–Wallis non-parametric test; results are reported in mean ± SD. Baseline ACTH levels at 0 h were compared with baseline ACTH levels at 2 h using a two tailed Mann–Whitney non-parametric test. For the acute response test we calculated the relative decrease of ACTH levels at 2, 3, 4, 5, and 6 h after a single s.c. pasireotide dose from the mean pre-dose ACTH levels (time points were at − 1, − 0.5 and 0 h before dose) as well as a mean relative decrease. Comparison of safety blood tests was with one-way ANOVA. The longitudinal data (ACTH levels) were analyzed using a linear mixed-random effects model. We report estimates for the coefficient(s) from these regression models along with their associated 95% confidence interval (CI). Tumor volumes before and after treatment were compared by paired t-test, assuming a normal distribution; results are reported in mean ± SD. A p value < 0.05 was considered statistically significant.

## Results

### Patients

Eight patients were recruited, all females. Of the eight patients, two withdrew during the s.c. phase (1, 8) and two in the LAR phase of treatment (5, 7) and 4 patients (2, 3, 4, 6) completed all of the study visits (Table 1). In all patients any radiotherapy had been administered at least 5 years prior to study entry. Patient 1 withdrew after 11 days of s.c. pasireotide 1200 μg b.d. due to abdominal cramps (resolved after stopping pasireotide). Patient 5 withdrew after completing the s.c. phase. Patient 7 withdrew during the LAR phase due to significant hyperglycemia that persisted at the end of the study visit 2 months after stopping pasireotide but improved to baseline on longer follow-up after study completion. Patient 8 withdrew during the first visit of the s.c. phase due to adverse events (felt unwell, drowsy, had a headache and was hypotensive during the visit); ACTH sampling from this visit was incomplete and therefore ACTH levels from this patient were not included in the statistical analysis.

**Table 1:** Summary of response to pasireotide treatment

Patient ID	Acute response test	s.c. phase			LAR phase		
	Relative decrease in plasma ACTH levels*	Daily dose (μg)	Treatment time	Response*	Monthly dose (mg)	Treatment time	Response*
1	–	1200	11 days	C	–	–	–
2	79%	1200	4 weeks	C	60	24 weeks	C
3	25%	600	4 weeks	C	40	24 weeks	No
4	84%	600	4 weeks	C	40	24 weeks	C
5	53%	1200	4 weeks	C	–	–	–
6	− 16%	1200	4 weeks	P	60	24 weeks	C
7	70%	1200	4 weeks	P	60	12 weeks	P
8	–	1200	0.5 day	No	–	–	–
Protocol	2-week	4-weeks			24-weeks		

*C* complete success, *P* partial success, *No* no success (for definitions please refer to Methods)

*Pre-test ACTH is the average of 3 time-points (− 1, − 0.5, 0 h) and post test average calculated from ACTH levels at 2, 3, 4, 5, and 6 h after 600 μg pasireotide s.c.

### Plasma ACTH levels improved during pasireotide treatment

**Fig. 2:**
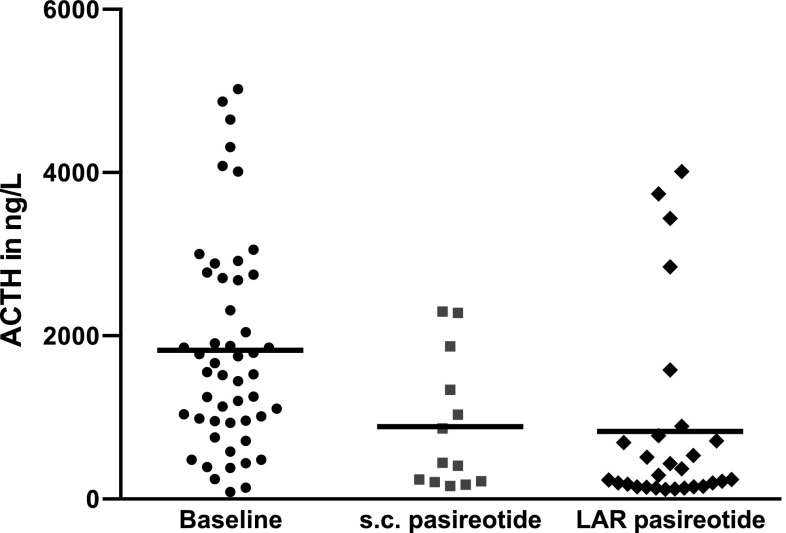
Mean plasma ACTH at 0 h prior to the morning dose of glucocorticoids improved during pasireotide treatment (mean baseline 1823 ± 1286 ng/l vs. 888.0 ± 812.8 ng/l during the s.c. phase and vs. 829.0 ± 1171 ng/l during the LAR phase, p < 0.0001)

ACTH levels at 0 h prior to the morning glucocorticoid dose (ACTH 0 h) at baseline were compared with ACTH 0 h levels during the s.c. phase and ACTH 0 h levels during the LAR phase. Overall, there was a significant reduction in ACTH 0 h during treatment (mean baseline 1823 ± 1286 ng/l vs. 888.0 ± 812.8 ng/l during the s.c. phase vs. 829.0 ± 1171 ng/l during the LAR phase, p < 0.0001, H = 20.93 mean ranks 57.3 vs. 37.5 vs. 29.8) (Fig. 2). Similarly, comparison of ACTH levels 2 h after the morning glucocorticoid dose showed reduction of ACTH 2 h levels during the two treatment phases (mean baseline 1100 ± 987 vs. 490.0 ± 460.3 ng/l during the s.c. phase vs. 262.2 ± 219.4 ng/l during the LAR phase, p = 0.001, H = 13.38 mean ranks 40.2 vs. 31.1 vs. 21.1). Baseline ACTH levels at 0 and 2 h post glucocorticoid dose were significantly different (p = 0.04). In order to investigate the change of ACTH levels over time, plasma ACTH (0 h pre-GC and 2 h post-GC) at each study visit and for each individual patient were analyzed using a marginal Generalized Linear Model (GLM) for longitudinal data. There was a statistically significant decline in the ACTH 0 h pre-GC levels throughout the study; ACTH 0 h levels declined by 26.1 ng/l per week (95% CI − 45.2 to − 7.1; p < 0.007). GLM analysis showed that plasma ACTH 2 h post-GC levels did not significantly change over time; ACTH 2 h levels declined by 4.0 ng/l per week, 95% CI − 12.58 to 4.49, p = 0.35.

**Fig. 3:**
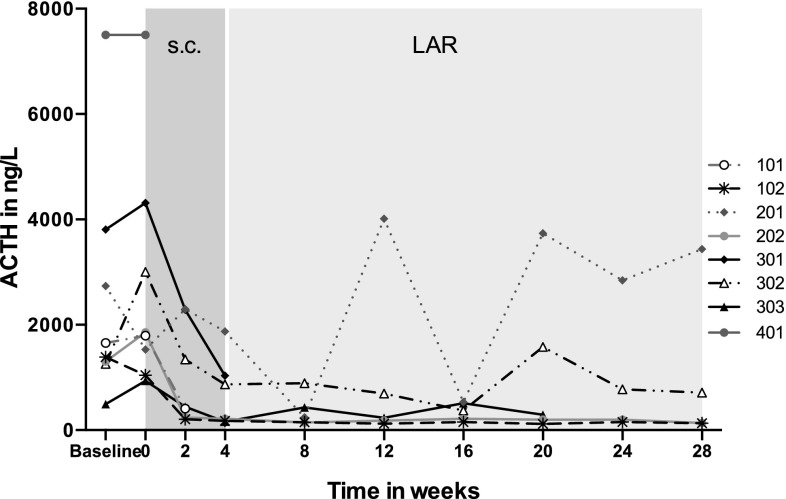
Individual plasma ACTH changes during the study in eight patients (ACTH levels before the morning dose of hydrocortisone)

Applying the *a priori* ACTH response criteria at the end of 4-week of s.c. pasireotide (or at the last visit if patient withdrew prior to the end of this phase) 5/8 patients had a complete response, 2/8 had a partial response while one patient did not respond (Table 1). The patient who did not respond withdrew very early from the study after one s.c. dose of pasireotide (patient 8). At the end of 24-week of pasireotide LAR treatment or at the last visit, 3/5 patients had a complete response, 1/5 a partial response and 1/5 showed no response. Four patients completed the study; 3/4 had a complete response at the end of the study and 1/4 did not respond (Table 1). Overall, 6/8 patients had complete or partial responses at their last biochemical assessment (either at the end of the study or last visit before withdrawal) (Fig. 3). There was no clear relationship between dose administered and effect.

### Acute response to pasireotide test dose

**Fig. 4:**
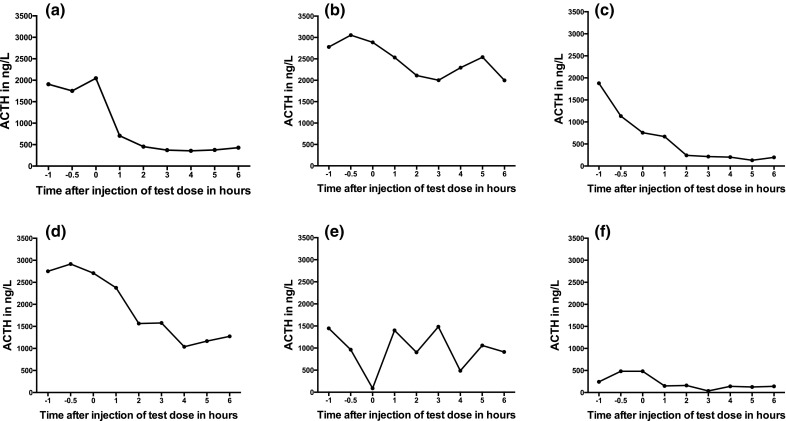
Acute response of plasma ACTH levels to a single dose of pasireotide 600 μg s.c. in 7 patients [Patients **a** 2, **b** 3, **c** 4, **d** 5, **e** 6, **f** 7]

Six patients received the pasireotide/placebo test dose while omitting their glucocorticoid treatment (Fig. 4); 5/6 patients showed a consistent reduction in plasma ACTH levels and one (patient 6) did not respond (patient 4 received her usual glucocorticoid dose during the test and is excluded). The mean relative decrease in plasma ACTH levels before and 2-6hours after a pasireotide test dose in the five patients who showed a positive response to the test was between 25 and 84% (patient 2—79%, patient 3—25%, patient 4—84%, patient 5—53%, patient 7—70%); all patients with a positive acute response (i.e. reduction in ACTH levels post pasireotide test dose) showed a positive response at the s.c. phase of treatment. The maximum reduction was observed between 4 and 6 h for all patients; those with a maximum relative decrease of at least 42% of their baseline ACTH levels following a test dose showed some response (complete or partial) to pasireotide treatment.

### Change in tumor volume and skin pigmentation

#### Tumor volume

Five patients had MRIs at screening and at the end of the study; four patients completed the 28-week of the treatment protocol (patients 2, 3, 4, 6) and one patient (patient 7) withdrew during the LAR phase. Overall, there was no significant change in tumor volumes between the pre-treatment and post-treatment scans (1.4 ± 0.9 vs. 1.3 ± 1.0, p = 0.86).

#### Skin pigmentation

There was no evidence of a change in skin pigmentation during the study as assessed by the independent assessor, although the attending physicians at the centers felt there was an improvement in 3 patients (patients 3, 6, 7).

### Hyperglycemia during treatment

**Fig. 5:**
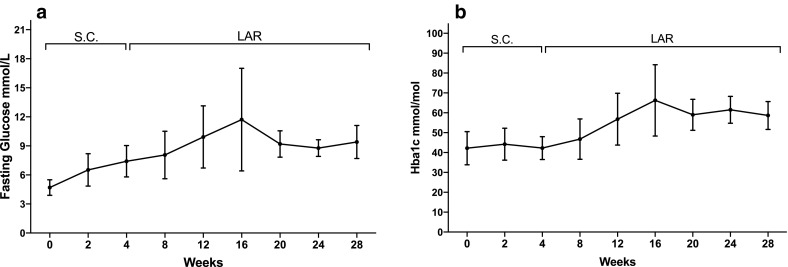
**a** Mean fasting glucose increased during pasireotide treatment (values from 7 patients included in the baseline mean value and s.c. phase, 5 patients for the LAR phase). **b** Mean HbA1c levels increased during pasireotide treatment (values from 7 patients during s.c. phase and 5 patients during LAR phase)

Fasting blood glucose and Hba1c increased during therapy and 6 patients developed hyperglycemia (Fig. 5). Fasting glucose: mean at baseline 4.6 ± 0.6 vs. 6.9 ± 1.6 mmol/l during s.c. phase vs. 9.6 ± 2.9 mmol/l during LAR phase, p < 0.01, Hba1c in mmol/mol: mean at baseline 42.9 ± 7.8 vs. 45.6 ± 8.5 during s.c. phase vs. 60.0 ± 13.6 during LAR phase, p < 0.01. Patient 7 withdrew from the study due to significant hyperglycemia after 16 weeks of treatment. Fasting insulin levels reduced during s.c. and LAR pasireotide treatment (mean baseline 118.1 ± 23.70 vs. mean during s.c. treatment 51.09 ± 12.52 vs. 64.94 ± 111.90 during LAR phase, p = 0.04).

### Adverse events

During the study the majority of patients reported diarrhea (7 patients), nausea and headaches (6 patients), dizziness (5), abdominal cramps (4), flu-like symptoms (4) and symptoms of hyperglycemia (4). There were no events attributed to cholelithiasis, no clinically significant events relating to baseline blood tests (electrolytes, renal and liver function tests).

## Discussion

Nelson’s syndrome affects a significant number of patients treated with bilateral adrenalectomy for the management of hypercortisolism associated with Cushing’s disease, and can be severely debilitating and life threatening. Although Nelson’s syndrome is a condition that may be anticipated to occur after BLA, it poses a significant clinical management challenge, as there is currently no medical treatment that works consistently. In this prospective clinical study we have shown that pasireotide significantly reduces plasma ACTH levels in patients with Nelson’s syndrome. All 7 patients treated with s.c. pasireotide (600 or 300 μg b.d.) had a significant reduction in plasma ACTH levels and 4 out of 5 patients who progressed to receive monthly LAR pasireotide treatment continued to show a biochemical response. Pasireotide could, therefore, be considered for the treatment of patients with Nelson’s syndrome especially if there is positive biochemical response to s.c. pasireotide after a short 4-week treatment trial. Following this period patients that respond could continue on s.c. treatment or change to monthly LAR administration.

In this 28-week study there were no conclusive changes in either skin pigmentation or tumor volume, although there was an indication of possible improvement in pigmentation in 3/7 patients and there was at least one patient with minimal improvement in tumor volume. It is reasonable to anticipate that a biochemical response would be followed by a reduction in tumor volume on long-term treatment and our negative findings could be due to the small patient numbers or the short duration of treatment. A reduction of tumor volume with pasireotide treatment has been documented in patients with CD [24, 32] and a case report of a patient with Nelson’s syndrome treated with pasireotide LAR [27]; similar to our findings, the reduction in ACTH in this case report was evident early, within 1 month of treatment with improvement of skin hyperpigmentation. Tumor shrinkage is also well documented in patients with acromegaly treated with first and second generation somatostatin analogs [33]. A longer period of treatment is needed to fully assess the effects of pasireotide on tumor volume in Nelson’s syndrome.

In the advent of personalized medicine, predicting which patients are more likely to benefit from pasireotide treatment is extremely desirable as it could avoid expensive unnecessary treatment trials and exposure of patients to potential side effects. A positive acute response to pasireotide test dose (i.e. reduction in plasma ACTH levels following a single 600 μg s.c. dose) may predict response to long-term treatment in the majority of patients, but a negative response does not exclude that a response will be seen; 5 out of 6 patients who had a consistent reduction in plasma ACTH after a test dose had a response to pasireotide treatment. Furthermore, patients that exhibited a decrease of plasma ACTH by at least 42% from baseline 4 to 6 h after a pasireotide test dose showed some response (partial or complete) to pasireotide treatment. Histopathological analysis of tissue samples in patients with prior pituitary surgery looking specifically at the expression of somatostatin receptors (SSTR) could be assessed as a factor for predicting responsiveness. Unfortunately the historical histological samples in this study were not available for re-examination but correlation of the SSTR expression patterns and biochemical response would have been interesting to examine and could help explain the differences in response between patients. However, it is also possible that SSTR expression from the original corticotroph tumor is different than the active Nelson’s tumor, and potentially this might be affected by other modalities of therapy, including radiotherapy. Furthermore, recent molecular studies in patients with CD suggest that there is enhanced SSTR5 mRNA expression in corticotroph adenomas harboring somatic mutations of the *USP8* gene, and it is possible that the presence of *USP8* mutations could help predict response to pasireotide treatment in Nelson’s tumors [34]. In our study no clear dose response relationship was observed on the effect on plasma ACTH. This may be due to varying expression of somatostatin receptors in the tumors or their signaling. Interestingly, there does not appear to be a dose response relationship for the effects of pasireotide in Cushing’s disease.

The future place of pasireotide in patients with Nelson’s syndrome needs to be balanced by its side effects, especially hyperglycemia. Hyperglycemia was a frequent adverse event associated with pasireotide treatment in this study with six out of seven patients developing abnormal fasting glucose and either new or worsening diabetes. Fasting glucose and HbA1c continued to increase during treatment in spite of the clinicians’ attempts to treat this medically and one patient withdrew due to hyperglycemia. Similarly high rates of hyperglycemia were reported in 49% of patients treated with LAR pasireotide [35] and 73% of patients treated with s.c. pasireotide (1200 or 1800 μg daily) for CD [24]; in this study 6% of patients discontinued treatment due to a hyperglycemia related adverse event and 46% had to start a new anti-diabetic medication. The significant fall in insulin levels observed in our study is consistent with suppression of insulin secretion from beta cells of the pancreas, in keeping with the known action of pasireotide at the somatostatin subtype 5 receptors on these cells [36]. The observed hyperglycemia following pasireotide treatment is due to the suppression of insulin and incretin response (glucagon-like peptide 1 and glucose-dependent insulinotropic polypeptide) [36]. Greater physician awareness of the pasireotide-associated hyperglycemia and more aggressive management of glucose-related AEs may make pasireotide more acceptable for managing this challenging condition [37]. Active monitoring and management of glucose homeostasis is needed and patients counseled about this prior to therapy.

The main limitations of this study are the small patient numbers, with this reflecting on overall generalizability, and the fact that half the patients did not complete the study. Although the recruitment target was not met, the results confirm a statistically significant biochemical effect even in this small patient size. Plasma ACTH levels before the morning administration of glucocorticoid dose (ACTH 0 h) are most commonly used for monitoring of patients with Nelson’s syndrome and our results show statistically significant reductions during treatment with the robust GLM test. A non-significant trend of reduction of plasma ACTH levels 2 h post glucocorticoid dose with GLM is likely due to lack of power and lower baseline levels of ACTH after glucocorticoid administration (mean baseline ACTH 0 h 1823 ± 1286 ng/l vs. mean baseline ACTH 2 h 1100 ± 987 ng/l). Three of the patients who showed response received radiation therapy 6–16 years prior to study entry and in the absence of historic ACTH levels a small lasting effect of radiation treatment on ACTH levels cannot be definitely excluded, but given the rapid fall in ACTH seen on treatment and the very long time period from radiation administration a significant contributing effect of radiation is unlikely. Treatment for periods longer than this study protocol (7 months) are likely needed to investigate the effect of treatment in tumor volume. The strengths of the study lie in the prospective design and the statistically significant evidence of biochemical response to medical therapy.

In conclusion, pasireotide treatment (s.c. and LAR) was effective in reducing ACTH levels in Nelson’s syndrome and might represent a potential treatment on an individualized basis as treatment options are limited; the lack of complete consistency of response precludes making firm recommendations. If considered, active monitoring and management of glucose homeostasis is mandatory. The patients who responded did so soon after initiation of pasireotide, and thus it would be reasonable to consider a complete lack of response after 2 months of treatment as a failure of response and therapy be discontinued. The LAR preparation appears as effective as the s.c. preparation and is likely to be more acceptable to patients. It would seem reasonable to commence therapy at a lower dose and escalate if tolerated, as there appears to be no clear relationship between dose and effect. Our study is limited, however, by the small sample size and duration of therapy, precluding wide generalizability, and further studies are needed of longer duration (12–24 months) in greater numbers to formally assess the impact of pasireotide in Nelson’s syndrome.

## Electronic supplementary material

Below is the link to the electronic supplementary material.

Supplementary material 1 (DOCX 120 KB)
