# Supplementary material for: A prospective longitudinal study of Pasireotide in Nelson’s syndrome
Source: Pituitary. 2018 Jan 8;21(3):247–55. doi: 10.1007/s11102-017-0853-3 (PMC5942349; doi:10.1007/s11102-017-0853-3)
Supplement: Supplementary file 1 — Supplementary material 1 (DOCX 120 KB) [file 11102_2017_853_MOESM1_ESM.docx]

**Supplemental Table: Patient characteristics**

| Patients | Age | Treatment for CD/ NS | Past medical history | Medications | Clinical examination | Biochemistry | Patients’ pathway |
| --- | --- | --- | --- | --- | --- | --- | --- |
| Patient 1 | 46 | TSS (1992 and 1998)  BAL (2003) | DVT (2002), cholecystectomy (1995), migraines, sinusitis | Hydrocortisone 10mg b.d., fludrocortisone 50 μg o.d., omeprazole 20mg o.d., atorvastatin 20mg o.d., levothyroxine 125 μg o.d., pseudoephedrine spray | Skin hyperpigmentation (shins, knuckles, chin, tongue, face), BMI 28, normotensive | ACTH* 1520ng/l, fasting blood sugar 4.5mmol/l, Hba1c 58mmol/mol, U&Es and LFTs were normal | Did not have test dose (but had baseline blood tests), withdrew during the s.c. phase (included in the ACTH efficacy analysis for 0h and 2h post HC for the s.c. phase only) |
| Patient 2 | 48 | TSS (1995), pituitary radiotherapy (1995), BAL | Psoriatic arthropathy (2008), hypertension, palpitations and irregular heart beat (2004), partial 3rd nerve palsy (2011) | Prednisolone (5mg/ 2.5mg), fludrocortisone 100μg, atenolol 25mg, amitriptyline 50mg, levothyroxine 100μg, adcalD3 1 b.d., methotrexate 15mg weekly, folic acid 5mg, simvastatin 40mg, ranitidine 150mg, mirapexin 125mg, growth hormone 0.5mg, progesterone only pill, and furosemide 20mg | Skin hyperpigmentation, normotensive, BMI 22.2  LFTs | ACTH* >1250ng/l, normal fasting blood sugar (4.8mmol/l), elevated HbA1c (50mmol/mol), normal U&Es and LFTs | Completed all visits |
| Patient 3 | 43 | TSS (1995), BAL (1999) | Previous cholecystectomy (1999) | Hydrocortisone (10/5/5mg), fludrocortisone 100μg bid, cyclizine 50mg t.d.s. p.r.n., loperamide 2mg p.r.n., hyoscine 10-20mg p.r.n., folic acid 5mg, ferrous sulphate 200mg o.d | Skin hyperpigmentation, hypotensive (88/55), BMI 25.1 | ACTH* 4872.0ng/l, normal fasting blood sugar (4.5mmol/l), HbA1c (40mmol/mol), U&Es and LFTs | Completed all study visits. Received lower dose of pasireotide (600μg daily during s.c. phase and 40mg monthly during LAR phase) due to poor tolerance |
| Patient 4 | 47 | TSS (1994), pituitary radiotherapy (1994), BAL (1998), stereotactic radiotherapy (2001) | Migraines (2001), hysterectomy (2011) | Hydrocortisone (10/5/5mg), fludrocortisone 100μg, growth hormone 0.2/0.1mg on alternative days o.d., sumitriptan 50mg p.r.n. | Skin hyperpigmentation, normotensive, BMI 31.5 | ACTH* 1854ng/l, normal fasting blood sugar (4.1mmol/l), HbA1c (32mmol/mol),U&Es and LFTs | Completed all study visits. Dose of pasireotide was reduced during the s.c. phase due to poor tolerance (to 600μg daily) and received lower dose (40mg monthly) during LAR phase |
| Patient 5 | 53 | TSS (1992), BAL (1983) | Hysterectomy for uterine malignancy (1987), cholecystectomy (1986), right nephrectomy (1988), thyroidectomy for goiter (1990), hypoparathyroidism, tachycardia (1995), shortness of breath, TIAs (1996), depression | Hydrocortisone (5/5mg), fludrocortisone 50μg o.d., fluconazole inhaler 250μg, levothyroxine 100μg o.d., omeprazole 20mg o.d., agomelatine 50mg o.d., alphacalciferol 75μg o.d., atorvastatin 10mg o.d., dipyridamole/aspirin 200/25mg o.d | Skin hyperpigmentation, normotensive, BMI 33.2 | ACTH* 4080.0ng/l, fasting blood sugar 5.8mmol/l, HbA1c 5.9%, Urea 13.7mmol/l, creatinine 140μmol/l normal LFTs | Withdrew after completing the s.c. phase and was not included in the efficacy analysis for the LAR phase |
| Patient 6 | 62 | TSS (1995), g- knife radiosurgery (2007), BAL (2013) | Osteoporosis, fractured pubic ramus (2013), headaches | Hydrocortisone (10/5/5mg), fludrocortisone 150μgo.d., alendronic acid 70mg weekly, adcal-D3 b.d., levothyroxine 50μg, growth hormone 0.5mg o.d., ibuprofen 200mg p.r.n. | Widespread skin hyperpigmentation, symmetrical muscle weakness, normotensive, BMI 21.2 | ACTH* 142.0ng/l, normal fasting blood sugar (3.9mmol/l), HbA1c (37mmol/mol), electrolytes and LFTs were normal, creatinine (114μmol/l) | Completed all visits |
| Patient 7 | 62 | TSS (2007, 2010), BAL (2011) | Hysterectomy (1996), hypertension, cholecystectomy (2003) | Hydrocortisone (10/10/5mg), fludrocortisone 50/100μg on alternative days, losartan 25mg, levothyroxine 100μg, pravastatin 10mg, fluoxetine 100mg | Skin hyperpigmentation, normotension, BMI 34.0 | ACTH* 381.0ng/l, normal fasting blood sugar (3.4mmol/l), HbA1c (45mmol/mol), U&Es and LFTs | Withdrew during LAR phase due to significant hyperglycemia |
| Patient 8 | 59 | Radiotherapy, BAL |  | Hydrocortisone (20/10mg), fludrocortisone 100μg | Skin hyperpigmentation, normotensive, BMI 23.0 | ACTH* >7500ng/l, normal fasting blood glucose (4.6mmol/l), HbA1c (6%), U&Es and LFTs | Withdrew early in the s.c. phase (only included in the efficacy analysis for the 2h post-HC ACTH levels for the s.c. phase) |
| CD: Cushing’s disease, NS: Nelson’s syndrome, BAL: bilateral adrenalectomy, ACTH*=plasma ACTH levels prior to the morning dose of glucocorticoid, U&Es: urea, creatinine, sodium and potassium, LFTs: liver function tests, Hba1c: glycated hemoglobin | | | | | | | |
